# Sonic Hedgehog and Triiodothyronine Pathway Interact in Mouse Embryonic Neural Stem Cells

**DOI:** 10.3390/ijms21103672

**Published:** 2020-05-23

**Authors:** Pavel Ostasov, Jan Tuma, Pavel Pitule, Jiri Moravec, Zbynek Houdek, Frantisek Vozeh, Milena Kralickova, Jan Cendelin, Vaclav Babuska

**Affiliations:** 1 Laboratory of Tumor Biology and Immunotherapy, Biomedical Center, Faculty of Medicine in Pilsen, Charles University, alej Svobody 1655/76, 323 00 Plzen, Czech Republic; pavel.ostasov@lfp.cuni.cz (P.O.); pavel.pitule@lfp.cuni.cz (P.P.); 2 Department of Histology and Embryology, Faculty of Medicine in Pilsen, Charles University, Karlovarska 48, 301 66 Plzen, Czech Republic; milena.kralickova@lfp.cuni.cz; 3 Department of Pathophysiology, Faculty of Medicine in Pilsen, Charles University, alej Svobody 1655/76, 323 00 Plzen, Czech Republic; jan.tuma@lfp.cuni.cz (J.T.); zbynek.houdek@lfp.cuni.cz (Z.H.); frantisek.vozeh@lfp.cuni.cz (F.V.); jan.cendelin@lfp.cuni.cz (J.C.); 4 Laboratory of Neurodegenerative Disorders, Biomedical Center, Faculty of Medicine in Pilsen, Charles University, alej Svobody 1655/76, 323 00 Plzen, Czech Republic; 5 Laboratory of Proteomics, Biomedical Center, Faculty of Medicine in Pilsen, Charles University, alej Svobody 1655/76, 323 00 Plzen, Czech Republic; jiri.moravec@lfp.cuni.cz; 6 Department of Biology, Faculty of Medicine in Pilsen, Charles University, alej Svobody 1655/76, 323 00 Plzen, Czech Republic; 7 Department of Medical Chemistry and Biochemistry, Faculty of Medicine in Pilsen, Charles University, Karlovarska 48, 301 66 Plzen, Czech Republic

**Keywords:** cell differentiation, embryonic neural stem cells, sonic hedgehog, triiodothyronine

## Abstract

Neural stem cells are fundamental to development of the central nervous system (CNS)—as well as its plasticity and regeneration—and represent a potential tool for neuro transplantation therapy and research. This study is focused on examination of the proliferation dynamic and fate of embryonic neural stem cells (eNSCs) under differentiating conditions. In this work, we analyzed eNSCs differentiating alone and in the presence of sonic hedgehog (SHH) or triiodothyronine (T3) which play an important role in the development of the CNS. We found that inhibition of the SHH pathway and activation of the T3 pathway increased cellular health and survival of differentiating eNSCs. In addition, T3 was able to increase the expression of the gene for the receptor smoothened (*Smo*), which is part of the SHH signaling cascade, while SHH increased the expression of the T3 receptor beta gene (*Thrb*). This might be the reason why the combination of SHH and T3 increased the expression of the thyroxine 5-deiodinase type III gene (*Dio3*), which inhibits T3 activity, which in turn affects cellular health and proliferation activity of eNSCs.

## 1. Introduction

Embryonic neural stem cells (eNSCs) give rise to almost all cell types found in the central nervous system (CNS), thus they play an important role in brain development. Neural stem cells are also a source of postnatal neurogenesis which is one of the mechanisms of brain plasticity and regeneration in the adult brain. Thus, eNSCs represent a useful tool for development of neurotransplantation therapy for neurological diseases. The fate of both endogenous and grafted eNSCs is regulated by many factors that control their survival, proliferation and differentiation. A lack or excess of these factors could adversely affect brain development or function. On the other hand, targeted modulation of the level of these information molecules might have therapeutic value in some neurological diseases or might be used to pre-treat the cells in vitro before engraftment to enhance their survival and ensure adequate differentiation which still hinders the development of successful transplantation therapies [1,2,3]. In this work, we analyzed eNSCs differentiating alone and in the presence of two important factors, sonic hedgehog and triiodothyronine.

Sonic hedgehog (SHH) and thyroid hormones (THs), especially triiodothyronine (T3), are known to influence eNSCs and have been shown to be indispensable for proper brain formation and development. Interestingly, these two factors have been already proposed to interact through the regulation of their respective pathways [4,5,6,7]. Thus, it is also possible that this interaction might be involved in neuronal development and may exert favorable effects on differentiating eNSCs in adult CNS.

T3 plays a crucial role in differentiation and growth of many organs, including the brain. Deficiency of THs during critical periods of brain development results in severe neurological damage and cretinism syndrome [8]. Experimentally induced developmental hypothyroidism in animal models revealed a widespread effect of THs on neural stem cells (NSCs) proliferation, migration, differentiation, axonal outgrowth and guidance, synaptogenesis and myelination [9,10,11,12,13,14]. For review see [15]. Although the role of THs is highlighted predominantly in embryonic/fetal brain development, they are known to enhance survival of neurons exposed to hypoxia. They also participate in neurogenesis and glial survival, as well as in interactions with neurotransmitter systems in the adult brain [16,17,18,19,20,21]. Desouza et al. showed that T3, the most potent thyroid hormone, also regulates expression of a key morphogenic factor SHH and its transmembrane co-receptors patched (PTCH1, *Ptch1*) and smoothened (SMO, *Smo*) in the adult brain [6].

SHH is an extracellular signaling protein that mediates fundamental processes in embryonic development [22]. SHH has been recognized as a mitogen, regulating proliferation and survival of NSCs both in embryonic and adult brains [23,24,25,26]. It has also been demonstrated that intra-cerebral administration of SHH can induce de novo neurogenesis in the predominantly non-neurogenic neocortex of adult mice [27]. The main receptor for SHH is a transmembrane protein PTCH1. In the absence of its ligand, PTCH1 probably functions as a channel for steroid molecules, subsequently inhibiting the seven-transmembrane receptor SMO [28]. When occupied by SHH, it relieves the inhibition of SMO, which is subsequently internalized and transported into the primary cilia, where the SMO induces expression of glioma-associated oncogene (GLI) transcription factors (GLI1, GLI2 and GLI3) or their switch from repressor to activator form [29]. One of the genes affected by this process is also thyroxine 5-deiodinase type III (DIO3, *Dio3*), one of the major T3- and T4-inactivating enzymes [30,31].

Reviewing all the above-mentioned reports, it is clear that eNSCs participate in CNS development in both the embryonic and adult brain and that this process is influenced by many factors including SHH and T3.

Therefore, in the present study we tried to understand the role of SHH and T3 on differentiation of early stage eNSCs which may provide new insights for regenerative medicine for neurological diseases using eNSCs. We analyzed several groups of genes, including SHH pathway-related genes, T3 pathway-related genes and genes specific to differentiated neuronal or glial cells and stem cells. We also measured cellular health (we use cellular health here as a hyponym for cell viability and metabolic/respiratory activity) by MTT viability assay, analyzed proliferation and differentiation of eNSCs and proteins released into the media during their differentiation. To assess whether the effects of T3 are mediated by the SHH signaling pathway, we used an SMO selective antagonist PF-5274857 [32]. eNSCs were analyzed shortly after the induction of the differentiation process. During this short period, eNSCs change from self-renewal to non-replicable highly specialized cells and could be very sensitive to SHH/T3 dysregulation.

## 2. Results

### 2.1. SHH and T3 Signaling Changes the Ability of eNSCs to Differentiate into Neurons and Glia

**Figure 1:**
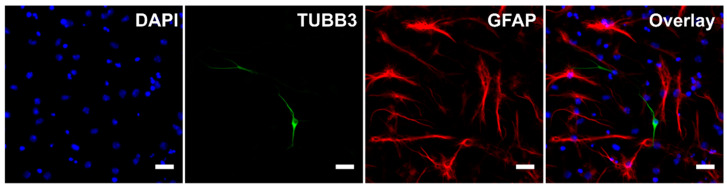
Immunocytochemistry. Representative image of cells after five days of differentiation without any treatment. Scale bar represents 20 µm. For the purpose of this image, gamma for GFAP channel was adjusted.

**Figure 2:**
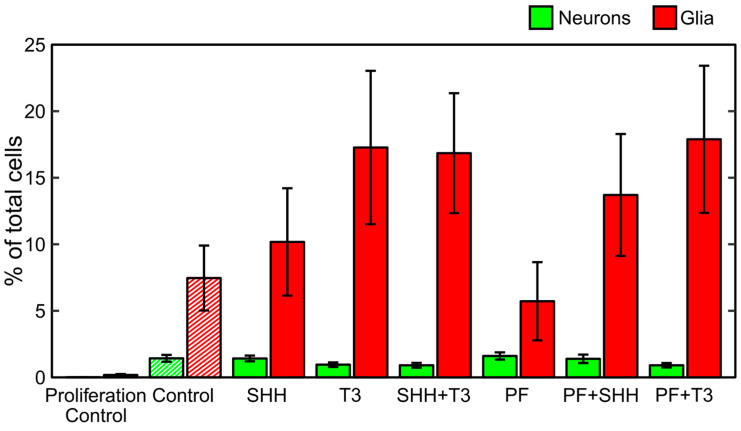
Differentiation analysis. Fraction of neurons and glia determined as neuronal (TUBB3-) and glial (GFAP-)positive cells detected on the last day of the differentiation phase of the experiment (DIV 18). The data represents an average from six experiments and the error bars represent ± SEM.

During the differentiation process, untreated cells became adherent to the bottom of the wells and most of them developed processes (Figure 1). Immunocytochemistry confirmed the differentiation of eNSCs into both neuronal (beta III tubulin, TUBB3) and glial (glial fibrillary acidic protein, GFAP) lineages (Figure 1). GFAP-positive astrocytes seemed to be well developed and neurons showed prominent staining of their bodies and processes in all differentiating treatments. Among non-differentiating proliferating control cells only 0.01% of all cells showed expression of TUBB3 and 0.17% showed expression of GFAP (Figure 2). In all treatments involving differentiation, at least 0.91% and 5.71% of cells were TUBB3- and GFAP-positive, respectively (Figure 2). We did not detect any significant difference in fraction of TUBB3- or GFAP-positive cells between differentiating control and any of the treatments, while non-differentiating proliferation control had a significantly lower fraction of both TUBB3- (*p* < 0.01) and GFAP-positive (*p* < 0.05) cells than differentiating control (Figure 2).

**Figure 3:**
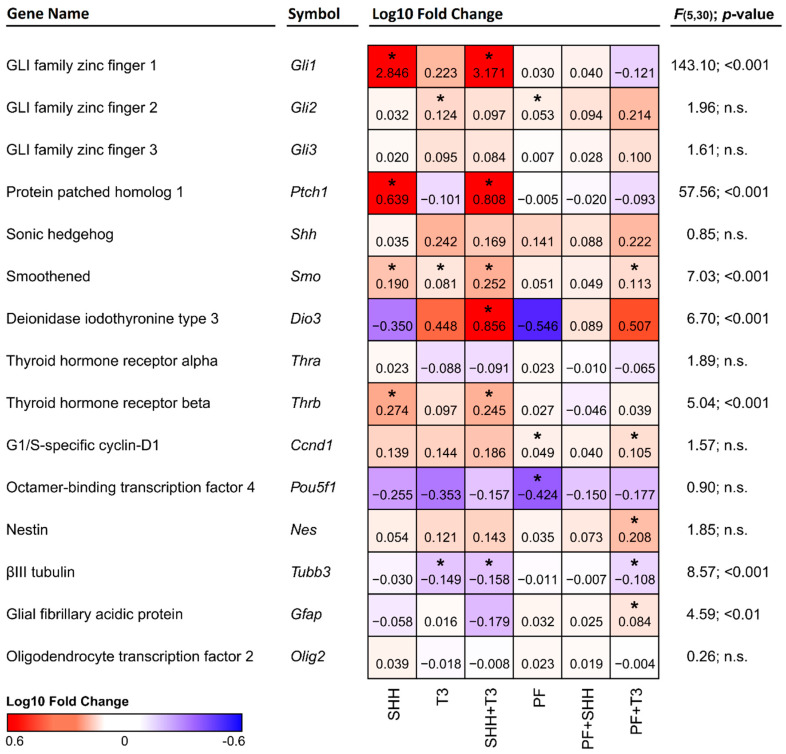
Heatmap depicting changes in the expression of individual genes relative to control cells. Red denotes an increase and blue denotes a decrease in expression. The numbers in the individual tiles represent average log10 fold change relative to control cells, and the asterisks indicate statistical significance (* *p* < 0.05). F and p show statistical significance of the relative effect of the treatment factor (permutation one-way ANOVA).

In analysis of individual treatments by qPCR, the *Gfap* gene was significantly upregulated (*p* < 0.05) when T3 was combined with the SHH pathway inhibitor PF-5274857. *Tubb3* gene expression was significantly suppressed (*p* < 0.05) in all cases where T3 was added (T3; SHH + T3; T3 + PF). This effect was not altered by SHH addition or inhibition by the SHH pathway inhibitor PF-5274857 (Figure 3). No marker of specific neuronal subtypes exhibited a significant change in expression with any treatment as depicted in Supplementary Figure S1.

### 2.2. SHH Signaling Inhibition Decreases Proliferation and Increases the Cellular Health of Differentiating eNSCs

**Figure 4:**
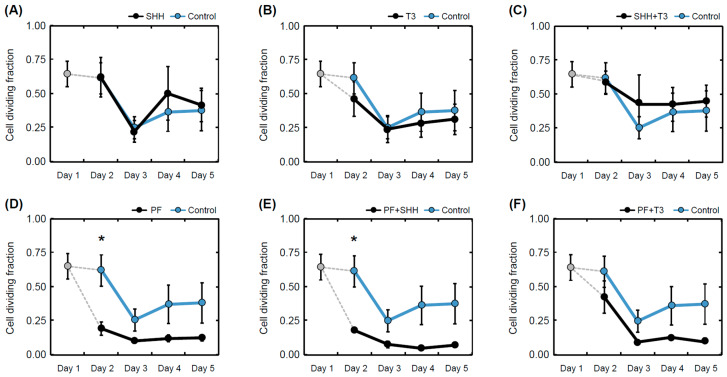
Mean proliferation rate. Proliferation rate determined as a fraction of dividing (BrdU-positive cell nuclei) cells over all detected cells in individual treatments (**A**–**F**) and controls on individual days of the differentiation phase of the experiment (day 1 to day 5). The error bars represent ± SEM. Statistically significant difference relative to control cells (between group): * *p* < 0.05. Statistical significance for within group comparisons (day 1 versus day 5) are provided in the text.

The cell proliferation rate during differentiation for each treatment was determined as the ratio of 5′-bromo-2-deoxy-uridine (BrdU)-positive cells to the total number of nuclei (Figure 4). The T3 hormone exposed that differentiating eNSCs significantly decreased their proliferation activity between day 1 and day 5 (within-group comparison, *p* < 0.05; Figure 4B). In the same manner, reduced proliferation was observed in cells exposed to treatments containing the SHH pathway inhibitor PF-5274857 (PF, *p* < 0.05; PF + SHH, *p* < 0.01; PF + T3, *p* < 0.05; Figure 4D–F). eNSCs exposed to PF-5274857 and PF-5274857 in combination with SHH also showed considerably decreased proliferative activity compared to the untreated control cells during the second day of the differentiation process (Figure 4D,E). However, in the following days, no significant differences compared with untreated control cells were observed (Figure 4).

**Figure 5:**
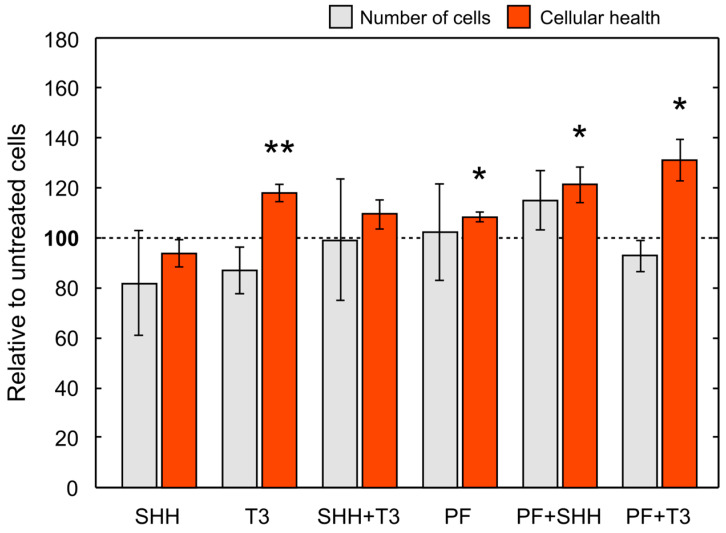
Mean cell number and cellular health. Cell number and cellular health as determined by the MTS test on the last day (day 5) of the differentiation phase of the experiment (i.e., DIV 18) relative to control cells. The error bars represent ± SEM. Statistically significant difference relative to control cells: * *p* < 0.05, ** *p* < 0.01.

Overall, cell numbers (data not shown) decreased during differentiation in all treatments with the exception of SHH and SHH in combination with T3 (untreated control, *p* < 0.01; T3, *p* < 0.001; PF, *p* < 0.001; PF + SHH, *p* < 0.01; PF + T3, *p* < 0.001). Initial cell numbers used for the differentiation phase were identical for all treatments as well as the control. For cell counts relative to untreated control on the last day of differentiation (day 5) see Figure 5. However, there were no significant differences in cell counts between individual treatments on day 5 (F_(5,65)_ = 0.99, *p* > 0.05; Figure 5). The cellular health and metabolic activity of eNSCs during differentiation were assessed using the MTS assay (Figure 5).

The significant effect of treatment administration on cellular health was revealed with one-way ANOVA (F_(5,43)_ = 4.91, *p* < 0.001). SHH administration did not change cellular health of differentiating eNSCs compared to untreated cells, while T3 exposure significantly increased eNSCs cellular health compared to both untreated control cells (*p* < 0.05) and SHH-treated cells (*p* < 0.01; Figure 5). Surprisingly, the inhibition of the SMO receptor using PF-5274857 significantly increased cellular health compared to untreated controls and this effect was also found in cells treated with PF-5274857 in combination with SHH or T3 (PF, *p* < 0.05; PF + SHH, *p* < 0.05; PF + T3, *p* < 0.05; Figure 5). Thus, all these SMO inhibitor-treated cells showed increased cellular health unlike SHH-treated cells.

### 2.3. SHH and T3 Alter the Expression of Each Other’s Pathway Components

The expression of the genes of interest (Figure 3) was analyzed by qPCR and compared in cells exposed to individual treatments and untreated control cells. Although we did not detect significant changes in the expression of *Shh* itself, a significant increase in *Ptch1* (*p* < 0.05) expression was detected in cells treated either with SHH alone or in combination with T3 (Figure 3). Overexpression of the *Ptch1* downstream activating transcription factor *Gli1* was also detected in the same treatments, while in almost all other treatments it remained barely detectable. This effect was specific to SHH, as it was suppressed in combination of PF-5274857 and SHH and was not present in T3 treated cells. The expression of another component of the SHH pathway, the receptor *Smo*, was enhanced by both SHH and T3 (*p* < 0.05 and *p* < 0.05, respectively). While their combination induced higher expression than T3 alone (*p* < 0.01), it was not significantly higher than with SHH alone. However, T3 was able to increase *Smo* expression even when SHH signaling was inhibited by PF-5274857. The last investigated member of the SHH signaling cascade *Gli3* was not significantly altered by any of the treatments.

Among analyzed components of the T3 signaling pathway (i.e., *Dio3*, *Thra* and *Thrb*), only T3 receptor alpha (*Thra*) gene did not show any significant change with any treatment. *Dio3* was significantly upregulated by the combination of SHH and T3 and T3 receptor beta (*Thrb*) gene was significantly upregulated by SHH either alone (*p* < 0.05) or in combination with T3 (*p* < 0.05), but not when the SHH signaling inhibitor PF-5274857 was present. It should also be noted that Fisher’s two-sample permutation test for location of *Ccnd1*, *Gli2*, *Nes* and *Pou5f1* (*Oct3/4*) indicated possible changes in expressions in specific treatments, however the differences between treatments were not supported by one-way ANOVA.

## 3. Discussion

In this work, we analyzed the effects of SHH and T3 pathways and their interactions on eNSCs differentiation, proliferation and survival under differentiating conditions. These factors not only have an impact on brain development and functioning via influencing eNSCs in vivo, but targeted modulation of their levels or activities could also potentially be used for regenerative neurotransplantation therapies. It could be beneficial to reduce cellular death and increase proliferation and generation of proper cell types in transplanted cells after grafting. In addition, understanding the effects of these factors and their crosstalk is necessary to mitigate risk of tumorigenesis or other undesired phenomena when manipulating signaling pathways in stem cells. While the effects of SHH and T3 on the proliferation and numbers of various CNS cell types have been reported in previous studies [5,6,7], we first tested how these factors affect eNSCs survival and proliferation under differentiating conditions.

Although the final number of cells in all treatments was not significantly different, T3 and the SHH inhibitor PF-5274857 increased the cellular health of differentiating eNSCs measured by the MTS assay. This suggests that enhanced T3 and/or suppressed SHH signaling might improve cellular health of the stem cells. In turn, this could potentially improve efficiency of in vitro culturing or help both intrinsic or extrinsic eNSCs cope with various critical conditions during pathological processes in the tissue or post-grafting period. Nevertheless, further studies are needed, particularly in vivo experiments to verify if such an effect is also relevant under the conditions of the living brain.

Regarding the nature of the MTS assay [33,34], it should be noted that the results are influenced by many factors and an increase in measured cellular health could be, among other mechanisms, caused by an increase in mitochondrial activity. SHH drives the fragmentation of mitochondria as well as a decrease in ATP production and oxygen consumption [35]. Thus, observed significant increase in eNSCs cellular health by PF-5274857, an inhibitor of the SHH signaling pathway, could hypothetically be due to prevention of such fragmentation. T3, on the other hand, increases the volume and activity of mitochondria by direct action on p43, the mitochondrial T3 receptor [36], which could potentially explain the similar increase in cellular health that was observed. This corresponds to the fact that addition of SHH abrogated this effect of T3. Since healthy mitochondria are usually important for survival of cells, both T3 addition and SHH pathway inhibition might be potentially beneficial for the long term survival of grafted cells. While the addition of PF-5274857 or T3 alone increased cellular health, the number of BrdU-positive cells strongly decreased compared to untreated controls. This result, in combination with similar final total cell numbers, might suggest that SHH signaling inhibition or T3 signaling activation increased survival of eNSCs during differentiation and simultaneously reduced cell proliferation. Such a combination of enhanced stem cell survival and reduced proliferation would be beneficial for proper graft survival and it could decrease the risk of malignancy development from these cells. Higher cell survival due to these treatments, although corresponding with their above mentioned effects on cellular health, would need verification and deeper analysis.

Overall, SHH and T3 seem to have opposing effects on cellular health and they could probably compete in terms of shaping mitochondrial physiology. Based on our results, we hypothesize that the T3 pathway activation or SHH pathway inhibition (rather than just absence), seems to be the most effective treatment examined in this study in improving cellular health of eNSCs in differentiating conditions.

Another goal was to identify if SHH and T3 could affect cellular type and eventually subtype into which eNSCs differentiate. The fact that eNSCs successfully differentiated was confirmed by immunocytochemistry which showed both cell lineages, TUBB3 and GFAP, expressing neurons and astrocytes, respectively. We observed discrete signs of the suppression of neuronal differentiation by T3, as it caused lower expression of *Tubb3* in all treatments where it was added. However, the immunocytochemistry did not indicate significant differences in the fraction of neurons between T3-treated cells and other treatments. On the other hand, when combined with the SHH inhibitor PF-5274857, T3 increased the expression of astrocyte marker *Gfap*, but this change did not appear to be connected with significant changes in GFAP-positive cell fraction. One potential explanation of the discrepancy in *Gfap* expression and GFAP-positive cell fraction might indicate astrocyte activation without expansion of their numbers. Activated astrocytes secrete a number of factors which increase cellular fitness and prevent cellular death [37], thus improving survival of neurons in an environment with many dying cells. We also tried to identify individual subtypes of neurons by qPCR in early stages, but we did not obtain any significant results and expression levels for their marker genes were lower than expected.

In the last part, we aimed to elucidate if the SHH and T3 pathways affect each other in differentiating eNSCs. Direct crosstalk between these pathways was already reported in keratinocytes, primary cortical neuron cultures and brain tissues [4,5,6,7]. However, it has not been previously tested whether these interactions occur during differentiation of eNSCs, which is a crucial process during the development of CNS, and whether it could be beneficial for survival and proper differentiation of these cells. In this work, we detected some indices for possible indirect interaction of SHH and T3 pathways at the mitochondrial level in eNSCs. One of the known mechanisms by which SHH influences T3-signaling is by enhancing expression of *Dio3*, a negative regulator of T3. This effect is mediated by GLI2 activation and has been described in keratinocytes [4]. However, we did not observe this effect in eNSCs when SHH was used alone, despite increased *Gli1* and *Ptch1* expression, which suggests GLI2 transcription factor activation on the protein level [38,39]. On the other hand, *Dio3* expression was significantly enhanced by T3 in combination with SHH, forming a negative feedback loop. Thus, it seems that the ability of T3 to induce *Dio3* expression in differentiating mouse eNSCs is enhanced by SHH pathway activation, which is in line with recently published results by Aw et al. [40] and Gil-Ibáñez et al. [41].

T3 enhanced the expression of *Smo* in the SHH pathway in an independent manner, as the combination of T3 and the SHH inhibitor PF-5274857 still yielded a significant increase in *Smo* expression. While we did not detect a significant effect of SHH addition on *Shh* gene expression, it significantly increased the expression of components of its own pathway, such as *Ptch1*, *Smo* and *Gli1*. In both treatments containing SHH without the inhibitor, expression of *Thrb*, a component of the T3 signaling pathway, increased. Data on the exact function of *Thrb* in eNSCs differentiation are scarce, but it seems to play an important role in the proliferation of neuroprogenitors in the development of the hippocampus [42] and cerebellum [43], oligodendrocyte differentiation [44] and cancer development [45]. As *Thrb* is often silenced or mutated in cancers [46], it seems that its increase by SHH might actually serve as part of an autoregulatory loop which prevents uncontrollable proliferation of cells. This could be important as malignancy initiated by grafted stem cells is one of the problems preventing further progress in cell transplantation therapy.

## 4. Materials and Methods

### 4.1. Animals

B6.BR and C57Bl/6J mice used to produce B6.BR × C57BL/6J F1 hybrid embryos were obtained from the Jackson Laboratories (Bar Harbor, ME, USA) and housed in a 12/12 h light/dark cycle with temperature 23 ± 1 °C and food and water ad libitum. All handling of experimental animals was performed in compliance with the EU Guidelines for Scientific Experimentation on Animals and with the permission of the Ethical Commission of the Faculty of Medicine in Pilsen (MSMT – 4016/2017-2; 14.2.2017, MŠMT – 608/2012 – 40, 4.1.2012; 397/2011-30, 12.1.2011).

### 4.2. Preparation, Cultivation and Differentiation of eNSCs

Donor females with conception-timed pregnancies were euthanized by overdose of thiopental (intraperitoneal administration) on embryonic day 12.5 (E12.5). Brains were dissected from embryos and eNSCs were prepared as follows. Isolated brains were mechanically disaggregated and digested in 1X Trypsin-EDTA (BioSera, Nuaille, FR) for 5 min at 37 °C. Trypsin was neutralized with DMEM/high glucose (GE Healthcare, Chicago, IL, USA) containing 20% fetal bovine serum (FBS; BioSera, Nuaille, Fr). To achieve single cell suspension, the cells were triturated and washed in DMEM/F12 (GE Healthcare, Chicago, IL) medium containing 2 mM L-glutamine (GE Healthcare, Chicago, IL), 1X insulin-transferrin-selenium (Gibco, Carlsbad, CA, USA) and 1% streptomycin/penicillin (GE Healthcare, Chicago, IL).

**Figure 6:**
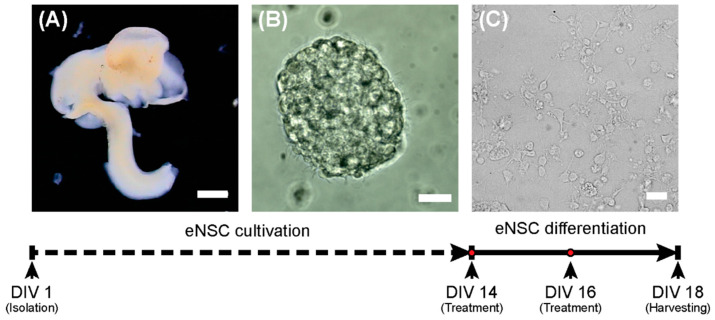
Scheme of the experiment. On the first day (DIV 1), eNSCs were harvested from the E12.5 embryonic central nervous system (**A**) and cultivated as neurospheres (**B**). On day 14 (DIV14), the differentiation process was started (**C**). Scale bars represent 1 mm for (**A**) and 20 µm (**B**,**C**).

The single cell suspension was seeded at 1 × 10^6^ cells/mL in NeuroCult^TM^ Proliferation medium (STEMCELL Technologies, Cambridge, UK) containing 20 ng/mL EGF (PeproTech, London, UK) and 1% streptomycin/penicillin (GE Healthcare, Chicago, IL). eNSCs were expanded for 14 days as floating neurospheres and passaged when they had reached approximately 150 μm in diameter. To induce the neural differentiation process, dissociated eNSCs were seeded on poly-L-ornithine (15 μg/mL; Sigma-Aldrich, St. Louis, MO) coated plates or glass cover slides at a density of 3 × 10^5^ cells/cm^2^ and cultured for 5 days in NeuroCult^TM^ differentiation medium (STEMCELL Technologies, Cambridge, UK) supplemented with 1% streptomycin/penicillin (GE Healthcare, Chicago, IL). The design of the experiment is schematically depicted in Figure 6.

### 4.3. Experimental Treatments

Recombinant mouse SHH (1 μg/mL; STEMCELL Technologies, Cambridge, UK), T3 (0.5 ng/mL; Sigma-Aldrich, St. Louis, MO) and the smoothened inhibitor PF-5274857 hydrochloride (PF; 100 nM; Sigma-Aldrich, St. Louis, MO), as well as their combinations (SHH + T3, SHH + PF, T3 + PF) in appropriate concentrations were added to differentiating cells at day 14 (DIV 14) and day 16 (DIV 16) in vitro (Figure 6). The concentrations of the compounds were derived from previous studies [32,47,48].

### 4.4. Measurement of Cellular Health and Proliferation

The proliferative capability of eNSCs in the course of the differentiation process was analyzed using BrdU immunolabeling [25,49]. To analyze eNSCs proliferation before the differentiation process, one portion of neurospheres was removed from the culture vessels on DIV 13, dissociated, seeded into 96-well plates and then cultivated for 24 h in proliferation medium (see above) containing 3 μg/mL of BrdU (Sigma-Aldrich, St. Louis, MO). The rest of the neurospheres were dissociated on DIV 14 and differentiated in four 96-well plates for up to 4 days. On each day of differentiation, one plate was exposed to BrdU (3 μg/mL) for 24 h and immediately fixed with 4% paraformaldehyde (PFA) in phosphate-buffered saline (PBS) for 15 min at 37 °C. BrdU immunostaining was performed according to a modified protocol described elsewhere [50,51]. Briefly, fixed cells were washed with Tris-buffered saline (Sigma-Aldrich, St. Louis, MO) (TBS), blocked in TBS containing 2% Triton X-100 (Sigma-Aldrich, St. Louis, MO) and 10% donkey serum (Abcam, Cambridge, UK) and treated with 2 M hydrochloric acid for 30 min at 37 °C for DNA denaturation. After this step, cells were blocked again and incubated with a polyclonal anti-BrdU primary antibody (1:500; cat. no ab1893; Abcam, Cambridge, UK) diluted in TBS containing 2% Triton X-100 and 1.5% donkey serum at 4 °C overnight. Thereafter, cells were washed in Tris-buffered saline and incubated with polyclonal DyLight^®^ 594 secondary antibody (1:400; cat. no ab96941; Abcam, Cambridge, UK) and diluted in the same buffer as the primary antibody for 4 h at room temperature. Subsequently, the cell nuclei were stained with DAPI (Sigma-Aldrich, St. Louis, MO) and immediately imaged using a Nikon Ti-E microscope equipped with a DS-Qi1Mc camera and a 20× plan fluorite objective at a 1280 × 1024 pixels resolution. Three fields were imaged from each well. Areas within individual nuclei in each image were then automatically selected based on DAPI fluorescence, and BrdU fluorescence intensity was measured in each area. For determination of the intensity that represented specific staining, BrdU fluorescence was determined in cells that had not been subjected to BrdU addition. The maximum fluorescence intensity recorded in these cells was defined as the threshold for determination of BrdU-positive staining. The total number of nuclei and the number of BrdU-positive nuclei was recorded. A total of three independent experiments with three replicates were performed. CellProfiler software was used for the identification of individual nuclei and image quantification.

Cell cellular health after 5 days of differentiation with individual treatments was also assessed using the CellTiter 96^®^ AQueous Non-Radioactive Cell Proliferation assay (MTS; Promega, Madison, WI). eNSCs were differentiated on 96-well plates for 5 days (see above) in six independent experiments with at least six replicates for each treatment. On the last day, the differentiation medium in each well was replaced with 120 µL of a mixture containing 100 µL of differentiation media and 20 µL of MTS reagent. After that, cells were incubated for 2.5 h and the absorbance was measured at 490 nm using Infinite^®^ M200 Pro (Tecan, Männedorf, CH) microplate reader.

### 4.5. Cell Immunostaining and Differentiation Analysis

Neural differentiation of eNSCs was verified using immunocytochemistry. The eNSCs were seeded on 12-well plates containing poly-L-ornithine (15 μg/mL) coated glass cover slips in duplicates and differentiated as described above. One duplicate was seeded in proliferation medium and fixed next day to serve as non-differentiating proliferation control. After 5 days of differentiation, cells were fixed with 4% PFA for 15 min at 37 °C and washed three times with PBS. Fixed cells were incubated with mouse monoclonal anti-beta III tubulin antibody [2G10] (TUBB3; 1:400; cat. no ab195879; Abcam, Cambridge, UK) conjugated with Alexa Fluor^®^ 488 and mouse monoclonal anti-glial fibrillary acidic protein [GA5] (GFAP; 1:400; cat. no. C9205; Sigma-Aldrich, St. Louis, MO) conjugated with Cy3^TM^ diluted in PBS containing 0.3% Triton X-100 (Sigma-Aldrich, St. Louis, MO) overnight at 4 °C. They were then mounted using Fluoroshield^TM^ with DAPI (cat. no. F6057; Sigma-Aldrich, St. Louis, MO). The samples were visualized using fully motorized Olympus IX83 microscope equipped with a 20× plan fluorite objective at resolution 1986 × 1454 pixels. From each slide, 12 non-overlapping fields were selected for analysis. Areas of individual nuclei in each image were then automatically selected based on DAPI fluorescence. Green (TUBB3) and red (GFAP) fluorescence intensity was measured in each area to determine numbers of neurons and glia in each field. CellProfiler software was used for identification of individual nuclei and image quantification [52]. For selection of positive signal threshold in automated analysis, GFAP-positive cells were manually counted in ten fields of differentiated eNSCs and the threshold giving the most similar results in automated analysis of these fields was used. Overall, three biological replicates were used for analysis, each consisting of a technical duplicate.

### 4.6. Gene Expression Analysis

For the analysis of gene expression, eNSCs were seeded into 6-well plates and differentiated according to the experimental protocol (see above) in six independent experiments. At the end of the differentiation process (DIV 18), the medium was collected for further proteomic analysis and cells were harvested by adding 700 μL of RNA Lysis Buffer (Zymo Research, Irvine, CA, USA) and stored at −80 °C until further processing. RNA was isolated using a Quick-RNA^TM^ MiniPrep (Zymo Research, Irvine, CA) according to the manufacturer’s instructions and its quality was assessed by agarose gel electrophoresis. cDNA was synthesized from 500 ng of total RNA in a 20 μL reaction volume using the RevertAid First Strand cDNA Synthesis Kit (Fermentas, Waltham, MA) according to the manufacturer’s protocol. The quality of cDNA and possible genomic DNA contamination was controlled by amplification of the reference gene *Hprt1* by qPCR. Expression of target and reference genes was assessed with TaqMan^®^ assays (probe information is listed in Supplementary Table S1) and TaqMan^®^ Gene Expression Master Mix using a 7500 Fast Real-Time PCR machine (Life Technologies, Carlsbad, CA, USA) with normalization to *Gapdh*, *Hprt1* and *Actb* expression. Ct values were obtained by 7500 software (ABI) and the threshold was set to 0.3 for all genes and experiments.

### 4.7. Proteomic Analysis

In addition, we also performed proteomic analysis of cell culture media to identify secreted factors and their changes. This additional experiment and its results are described in the supplementary file [53].

### 4.8. Statistical Analysis

The data from nuclei counts, MTS and gene expression expressed as relative values to control cells were analyzed using Fisher’s one-sample permutation test for location. The proliferative capability of eNSCs was evaluated using Fisher’s two-sample permutation test for location [54]. The effect of different treatments on cellular health measured by MTS and gene expression was analyzed using permutation one-way ANOVA followed by Fisher’s two-sample randomization test for location [54]. Both two-sample as well as one-sample Fisher’s permutation tests were performed with two-side alternative hypothesis. All permutation tests were performed with enumeration of all possible permutations. Reported F and t statistics were considered as F0, before the start of permutation. For analysis involving multiple comparisons, Benjamini–Hochberg correction for false discovery rate was used [55]. Data are presented as the mean ± SEM. A *p*-value < 0.05 was considered statistically significant. Statistical analyses were conducted using R with packages EnvStats 2.1.0 and lmPerm 1.1.

## 5. Conclusions

We identified that SHH signaling inhibitor PF-5274857 and T3 hormone increased cellular health of eNSCs, although proliferation rate was slightly suppressed which could be beneficial for survival of these cells after engraftment. We confirmed interaction between the SHH and T3 pathways in differentiating eNSCs. We showed the ability of SHH to enhance T3-induced expression of *Dio3* and the ability of T3 to induce expression of the *Smo* receptor, which is part of the SHH signaling pathway. As DIO3 transforms T3 into its inactive form, this effect seems to suppress T3 signaling, while T3-induced SMO expression enhances SHH signaling. To our knowledge, we are the first to detect upregulation of *Thrb* by SHH.

## Supplementary Materials

Supplementary materials can be found at https://www.mdpi.com/1422-0067/21/10/3672/s1.

## Abbreviations

BrdU	5′-bromo-2-deoxy-uridine
CNS	central nervous system
DIO3	thyroxine 5-deiodinase type III
eNSCs	embryonic neural stem cells
GFAP	glial fibrillary acidic protein
GLI	glioma-associated oncogene
NSCs	neural stem cells
PBS	phosphate-buffered saline
PFA	paraformaldehyde
PTCH1	patched
SHH	sonic hedgehog
SMO	smoothened
T3	triiodothyronine
TBS	Tris-buffered saline
THRA	T3 receptor alpha
THRB	T3 receptor beta
THs	Thyroid hormones
TUBB3	Beta III tubulin
