# Supplementary material for: Sonic Hedgehog and Triiodothyronine Pathway Interact in Mouse Embryonic Neural Stem Cells
Source: Int J Mol Sci. 2020 May 23;21(10):3672. doi: 10.3390/ijms21103672 (PMC7279276; doi:10.3390/ijms21103672)
Supplement: Supplementary file 1 [file ijms-21-03672-s001.zip › Ostasov_Supplementary_file.docx]

**Supplementary material**

**SHH pathway activation induces thyroid hormone receptor beta expression in embryonic neural stem cells**

Pavel Ostasov, Jan Tuma, Pavel Pitule, Jiri Moravec, Zbynek Houdek, Frantisek Vozeh, Milena Kralickova, Jan Cendelin and Vaclav Babuska

**Methods**

**Proteomic media analysis**

Differentiation media were collected on the last day of the differentiation process (see section on gene expression analysis) and lyophilized using the Lyoquest 85 Plus Eco (Telstar) lyophilizer. Samples of lyophilized media were dissolved in 3 mL of DPBS buffer and the solution was concentrated using Vivaspin 6 (5000 MWCO) ultrafiltration device. This procedure was repeated twice and the total protein concentration was measured by the BCA assay (Sigma-Aldrich) in the retentate. Then, 100 μg of protein in each sample were digested according to a protocol described by Agger et al. [s1]. All chemicals needed for in-solution digestion were purchased from Sigma-Aldrich.

Liquid chromatography-mass spectrometry (LC-MS) was performed using a NanoLC 425 UPLC system (Eksigent, Dublin, CA, USA) coupled to a Triple TOF 5600 (AB Sciex) apparatus. For information-dependent acquisition (IDA) experiments, the sample was first loaded onto a trap column (nanoLC Trap column ChromXP C18-CL 350 μm × 0.5 mm, 3 μm × 120 Å; Eksigent, Dublin, CA, USA) for 10 min at a flow rate of 1 μl/min. The flow was then directed in line with the nanoLC column 3C18-CL (75 μm × 15 cm, 3 μm × 120 Å; Eksigent, Dublin, CA, USA) at a flow rate of 300 nl/min, which is connected to the spray tip (PicoTip Emitter Silica Tip™ by New Objective, Woburn, MA, USA). The total step gradient time was set at 120 min. Mobile phases A (0.1% HCOOH, 2% ACN in water) and B (0.1% HCOOH in ACN) were used to create the separation gradient for eluting peptides in the column at an increasing concentration of solvent B as follows: 5% for 5 min, 5% to 40% for 50 min; 40% to 95% for 5 min; 95% for 5 min; followed by 95% to 5% for 5 min and 5% for 10 min. All data were acquired from the TripleTOF MS using Analyst TF 1.7 software (AB Sciex) in the IDA mode. Peptide profiling was performed using a mass range of 350-1250 Da mass range, followed by an MS/MS product ion scan from 100 to 1800 Da with the abundance threshold set at more than 120 cps. The accumulation time for ions was set at 100 ms. Target ions were excluded from the scan for 12 s after being detected and former ions were excluded from the scan after one repetition. The IDA advanced “rolling collision energy (CE)” option was employed to automatically ramp up the CE value in the collision cell as the m/z value was increased. A maximum of 25 spectra were collected from candidate ions per cycle. Two technical replicates were performed in the IDA experiments.

ProteinPilot^TM^ Software v4.5 (AB Sciex) was used for sample profiles with the Paragon method to source for possible peptide matches from the UniProt protein database (release2015_05). Search parameters in the Paragon algorithm in ProteinPilotTM Software were set as: Sample type: identification; Cys alkylation: Iodoacetamide; Digestion: trypsin; Instrument: TripleTOF 5600; Special Factors: none; Species: *Mus musculus*; Search Effort: Thorough ID. False discovery rate (FDR) analysis in the ProteinPilot^TM^ Software was performed and FDR < 1% was set for protein identification (based on ProteinPilot^TM^ Software and PSPEP, AB Sciex). Contaminants and reverse proteins identified through a database search were excluded from the analysis.

The subcellular localization of detected proteins was predicted by TargetP 1.1. The location assignment is based on the predicted presence of any of the N-terminal presequences: mitochondrial targeting peptide (mTP) or secretory pathway signal peptide (SP) [1].

**SDS-PAGE**

Samples were heated in sample buffer (4x Laemmli Sample Buffer, Bio-Rad) at 95°C for 5 min and loaded onto 12-well Mini-Protean™ TGX™ 4-15% Stain Free Gels (Bio-Rad). Electrophoresis was then performed in Tris/glycine/SDS running buffer at 250 V until the bromophenol blue dye front reached the bottom of the gel and imaged on ChemiDoc MP Imager (Bio-Rad).

**Western Blotting**

After electrophoresis the gel was immediately transferred to Trans-Blot® Turbo™ Mini PVDF Transfer Pack (Bio-Rad) and blotted on Trans-Blot® Turbo™ Transfer Starter System (Bio-Rad) in mixed MW settings. PVDF Membrane was scanned and blocked with 3% BSA in Tris Buffered Saline (TBS) and then was washed twice with TBS with Tween-20 solution (TBST) and incubated for 3 hours with anti-GAPDH antibody (SAB1405848, Sigma-Aldrich) diluted in TBST (1:500). The membrane was then washed twice with TBST and incubated with horse radish peroxidase-conjugated secondary antibody (Bio-Rad) diluted in TBST (1:500) for one hour. Signal was detected with ImmunBlot Opti-4CN System (Bio-Rad) substrate on ChemiDoc MP Imager (Bio-Rad). Image processing was accomplished with Image Lab Software v. 4.1 (Bio-Rad).

**Results**

Proteomic analysis of lyophilized cultivation media was performed to detect possible changes to the secretome of the cells. Detailed information of the LC-MS analysis results are listed in the supplementary files in Tables S2 and S3. Overall, 458 unique proteins were identified in all groups. Media from cells treated with the SHH pathway inhibitor PF-5274857 contained on average three to four times more proteins than media from all other groups.

SHH protein was detected in all media samples collected from treatments with added SHH (SHH, SHH+T3 and PF+SHH), demonstrating its presence throughout the experiment. There were no unique treatment-specific proteins detected in the media from treatments without PF-5274857 (SHH, T3, SHH+T3 and control). However, all these samples contained the protein antithrombin III (ANT3_MOUSE), which was not present in treatments with the inhibitor PF-5274857. Moreover, in all treatments containing the inhibitor PF-5274857, we found 20 unique proteins (Table S4) which were not present in any of treatments without this inhibitor. Of these proteins, only five were predicted to be secreted (Table S5). We confirmed presence of GAPDH one of the 20 proteins that were exclusively detected in media from inhibitor PF-5274857-treated cells by western blotting (Figure S2). Protein quantification indicated 0.16, 0.30 and 0.34 μg GAPDH per 100 μg of total protein from medium in PF+SHH, PF+T3 and PF respectively. This result agrees with LC-MS analysis of media.

**Figure S2.** Western blot of GAPDH.

Western blot of GAPDH. Lane GAPDH represents load of 0.2 μg of pure protein. Following six lanes represents media from samples untreated with inhibitor PF-5274857. Last three lanes show media with this inhibitor in which the GAPDH was detected by LC-MS.

| **Protein names** | **Accession name** | **Gene names** |
| --- | --- | --- |
| ATP synthase subunit alpha, mitochondrial | Q03265 | Atp5a1 |
| Alpha-enolase | P17182 | Eno1 |
| Glyceraldehyde-3-phosphate dehydrogenase | P16858 | Gapdh |
| Histone H1.5 | P43276 | Hist1h1b |
| High mobility group protein B1 | P63158 | Hmgb1 |
| Heterogeneous nuclear ribonucleoprotein K | P61979 | Hnrnpk |
| Heat shock protein HSP 90-beta | P11499 | Hsp90ab1 |
| Myristoylated alanine-rich C-kinase substrate | P26645 | Marcks |
| Nucleolin | P09405 | Ncl |
| Protein disulfide-isomerase A3 | P27773 | Pdia3 |
| Peptidyl-prolyl cis-trans isomerase B | P24369 | Ppib |
| Pleiotrophin | P63089 | Ptn |
| 60S ribosomal protein L22 | P67984 | Rpl22 |
| 60S acidic ribosomal protein P2 | P99027 | Rplp2 |
| 40S ribosomal protein S8 | P62242 | Rps8 |
| Protein SET | Q9EQU5 | Set |
| SPARC-related modular calcium-binding protein 1 | Q8BLY1 | Smoc1 |
| Serine/arginine-rich splicing factor 1 | Q6PDM2 | Srsf1 |
| Tenascin-R | Q8BYI9 | Tnr |
| Transitional endoplasmic reticulum ATPase | Q01853 | Vcp |

**Table S4.** Proteins detected exclusively in the PF-5274857-treated groups.

| **Name** | **Lenght** | **mTP** | **SP** | **Other** | **Loc** | **RC** |
| --- | --- | --- | --- | --- | --- | --- |
| Q03265 | 553 | 0.974 | 0.010 | 0.058 | M | 1 |
| P17182 | 434 | 0.245 | 0.041 | 0.800 |  | 3 |
| P16858 | 333 | 0.660 | 0.084 | 0.239 | M | 3 |
| P43276 | 223 | 0.081 | 0.036 | 0.948 |  | 1 |
| P63158 | 215 | 0.301 | 0.056 | 0.724 |  | 3 |
| P61979 | 463 | 0.059 | 0.060 | 0.955 |  | 1 |
| P11499 | 724 | 0.047 | 0.105 | 0.924 |  | 1 |
| P26645 | 309 | 0.244 | 0.037 | 0.795 |  | 3 |
| P09405 | 707 | 0.199 | 0.058 | 0.821 |  | 2 |
| P27773 | 505 | 0.063 | 0.969 | 0.013 | S | 1 |
| P24369 | 216 | 0.052 | 0.884 | 0.050 | S | 1 |
| P63089 | 168 | 0.222 | 0.813 | 0.016 | S | 3 |
| P67984 | 128 | 0.103 | 0.065 | 0.890 |  | 2 |
| P99027 | 115 | 0.218 | 0.238 | 0.441 |  | 4 |
| P62242 | 208 | 0.196 | 0.038 | 0.859 |  | 2 |
| Q9EQU5 | 289 | 0.480 | 0.049 | 0.524 |  | 5 |
| Q8BLY1 | 463 | 0.336 | 0.777 | 0.010 | S | 3 |
| Q6PDM2 | 248 | 0.131 | 0.050 | 0.892 |  | 2 |
| Q8BYI9 | 1358 | 0.026 | 0.659 | 0.565 | S | 5 |
| Q01853 | 806 | 0.066 | 0.039 | 0.950 |  | 1 |

no cutoffs; winner-takes-all (default)

**Table S5.** Prediction of subcellular localization of proteins detected exclusively in inhibitor PF-

5274857 containing groups by TargetP. Length – length of AA chain; mTP - mitochondrial targeting peptide; SP - secretory pathway signal peptide; Loc – location of protein (M means mitochondrial, S means secretory); RC – reliability class - RC=1 99%, RC=2 95%.

**Supplementary references**

s1. Agger, S.A.; Marney, L.C.; Hoofnagle, A.N. Simultaneous Quantification of Apolipoprotein A-I and Apolipoprotein B by Liquid-Chromatography-Multiple-Reaction-Monitoring Mass Spectrometry. *Clin Chem* **2010**, *56*, 1804 – 1813. doi: 10.1373/clinchem.2010.152264

(in original article under reference number 52)
