# Supplementary material for: Sonic Hedgehog and Triiodothyronine Pathway Interact in Mouse Embryonic Neural Stem Cells
Source: Int J Mol Sci. 2020 May 23;21(10):3672. doi: 10.3390/ijms21103672 (PMC7279276; doi:10.3390/ijms21103672)
Supplement: Supplementary file 1 [file ijms-21-03672-s001.zip › TableS1.pdf]

Gene Name	Symbol	Typical expression	Assays ID
GLI-Kruppel family member GLI1	*Gli1*	Shh pathway	Mm00494654_m1
GLI-Kruppel family member GLI2	*Gli2*	Shh pathway	Mm01293111_m1
GLI-Kruppel family member GLI3	*Gli3*	Shh pathway	Mm00492345_m1
Patched 1	*Ptch1*	Shh pathway	Mm00436026_m1
Sonic hedgehog	*Shh*	Shh pathway	Mm00436528_m1
Smoothened	*Smo*	Shh pathway	Mm01162710_m1
Deiodinase type III	*Dio3*	T3 signaling pathway	Mm00548953_s1
Thyroid hormone receptor alpha	*Thra*	T3 signaling pathway	Mm00579691_m1
Thyroid hormone receptor beta	*Thrb*	T3 signaling pathway	Mm00437044_m1
Cyclin D1	*Ccnd1*	Mitotic cell cycle	Mm00432359_m1
POU domain, class 5, transcription factor 1 (Oct-3/4)	*Pou5f1*	Stem cells	Mm03053917_g1
Nestin	*Nes*	CNS progenitor cells	Mm00450205_m1
Tubulin, beta 3 class III	*Tubb3*	Neurons	Mm00727586_s1
Glial fibrillary acidic protein	*Gfap*	Astrocytes	Mm01253033_m1
Oligodendrocyte transcription factor 2	*Olig2*	Oligodendroglia	Mm01210556_m1
Solute carrier family 6, member 3 (Dat1)	*Slc6a3*	Dopaminergic neurons	Mm00438388_m1
Glutamate decarboxylase 1	*Gad1*	GABAergic neurons	Mm00725661_s1
Tryptophan hydroxylase 1	*Tph1*	Serotonergic neurons	Mm01202614_m1
solute carrier family 17, member 7 (Vglut1)	*Slc17a7*	Glutamatergic neurons	Mm00812886_m1
solute carrier family 17, member 7 (Vglut2)	*Slc17a6*	Glutamatergic neurons	Mm00499876_m1
Gylceraldehyde 3-phosphate dehydrogenase	*Gapdh*	Reference gene	Mm99999915_g1
Hypoxanthine guanine phosphoribosyl transferase	*Hprt1*	Reference gene	Mm00446968_m1
Actin, beta	*Actb*	Reference gene	Mm00607939_s1

S1 Table. List of used genes and corresponding TaqMan® probes.
