# Supplementary figures and images for: Sonic Hedgehog and Triiodothyronine Pathway Interact in Mouse Embryonic Neural Stem Cells
Source: Int J Mol Sci. 2020 May 23;21(10):3672. doi: 10.3390/ijms21103672 (PMC7279276; doi:10.3390/ijms21103672)

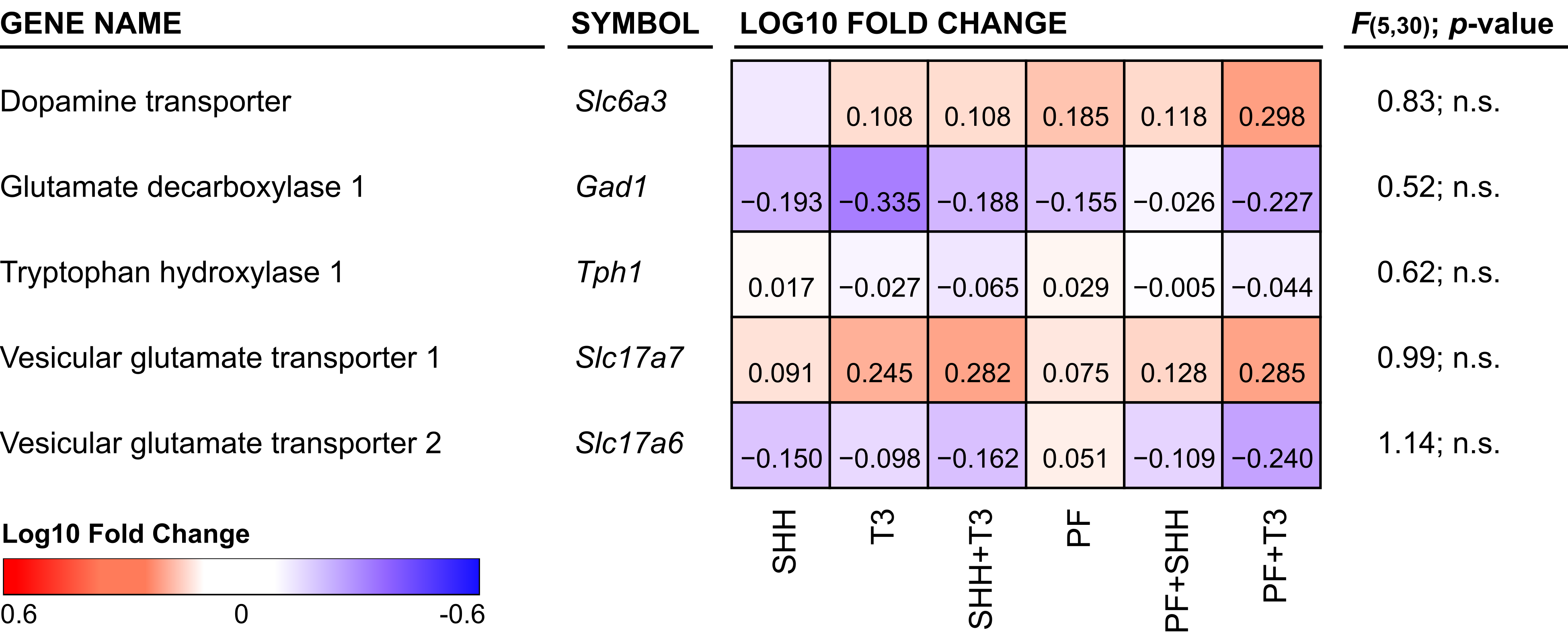

Supplement: Supplementary file 1 [file ijms-21-03672-s001.zip › FigureS1.tif]
